# Proteomic evaluation of sheep serum proteins

**DOI:** 10.1186/1746-6148-8-66

**Published:** 2012-05-25

**Authors:** Elisabetta Chiaradia, Luca Avellini, Micaela Tartaglia, Alberto Gaiti, Ingo Just, Fausto Scoppetta, Zoltan Czentnar, Andreas Pich

**Affiliations:** 1 Department of Pathologic, Diagnostic, and Clinical Veterinary Medicine, University of Perugia, Via San Costanzo 4, 06126, Perugia, Italy; 2 Institute of Toxicology, Hannover Medical School, Carl-Neuberg-Str. 1, 30625, Hannover, Germany

**Keywords:** Sheep, Serum markers, Proteomics, Two-dimensional electrophoresis, Proteins

## Abstract

**Background:**

The applications of proteomic strategies to ovine medicine remain limited. The definition of serum proteome may be a good tool to identify useful protein biomarkers for recognising sub-clinical conditions and overt disease in sheep. Findings from bovine species are often directly translated for use in ovine medicine. In order to characterize normal protein patterns and improve knowledge of molecular species-specific characteristics, we generated a two-dimensional reference map of sheep serum. The possible application of this approach was tested by analysing serum protein patterns in ewes with mild broncho-pulmonary disease, which is very common in sheep and in the peripartum period which is a stressful time, with a high incidence of infectious and parasitic diseases.

**Results:**

This study generated the first reference 2-DE maps of sheep serum. Overall, 250 protein spots were analyzed, and 138 identified.

Compared with healthy sheep, serum protein profiles of animals with rhino-tracheo-bronchitis showed a significant decrease in protein spots identified as transthyretin, apolipoprotein A1 and a significant increase in spots identified as haptoglobin, endopin 1b and alpha1B glycoprotein.

In the peripartum period, haptoglobin, alpha-1-acid glycoprotein, apolipoprotein A1 levels rose, while transthyretin content dropped.

**Conclusions:**

This study describes applications of proteomics in putative biomarker discovery for early diagnosis as well as for monitoring the physiological and metabolic situations critical for ovine welfare.

## Background

The application of proteomic approaches over the last decade has provided new tools for clarifying the molecular aspects of physiological states, and for understanding the etiology and pathogenesis of many diseases. Nevertheless, proteomic studies in veterinary medicine, particularly in the ovine species, remain limited compared with human medicine. Proteomics performs large-scale protein analysis, describes the entire protein complement of a cell, tissue, biological fluid or organism and provides information on protein expression, localization, functions and interactions. This approach could help in recognising sub-clinical conditions that compromise the product quality of an entire flock. For farm animals such as cows, sheep, and goats, the description of metabolic imbalances could serve to improve nutritional breeding management. If imbalances are not corrected they can lead to serious consequences in the food chain and financial losses.

Proteomics has been applied in studies on cows [1-4], horses [5], swine [6], chickens [7], and some domestic pets [8], to define the protein profiles of tissues and body fluids during stressful conditions, pathological states, and infections.

In the last decade proteomic technologies were successfully used in the discovery phase of biomarker identification [9], identifying many proteins that discriminated the “healthy” state from the “disease” states. This step has to be followed up by other phases that validate markers and prove and establish efficacy [10]. Knowledge of species-specific protein peculiarity is crucial in ovine medicine, as findings from bovine species are often directly translated for use in sheep. Characterization of serum and plasma proteomes has helped define new diagnostic and prognostic markers for humans and for a few other species [1-4,6,11-13].

The goals of this study were to define the serum sheep proteome and identify protein markers that recognize early and subclinical disease. A two-dimensional electrophoresis gel reference map of the sheep serum protein profile was generated as the first essential step. In addition, two applications of proteomic approaches in ovine medicine were tested. Serum protein changes were analysed in sheep during the peripartum period, and in animals with mild respiratory diseases.

Peripartum is a crucial period in the reproductive cycle of a lactating ruminant [14,15]. The metabolic and immunological status, feeding, and environmental conditions change rapidly and intensively [16]. In ewes, metabolic disorders and the risk of infections increase [17,18]. Inadequate management can affect the productivity and profitability of an animal or entire flock [14,15,18-21].

Respiratory diseases are commonly found in sheep flocks and can cause significant financial losses. In small ruminants, these diseases are often due to a combination of different causes such as viral and bacterial infections, physiological stress, poor management and environmental factors and also adverse weather conditions [22,23]. Diagnosis is often too late and based on post-mortem findings that need to be confirmed by serological markers.

 We analysed serum from sheep with rhino-tracheo-bronchitis or in peripartum status to identify candidate markers that could be used to indicate the need for immediate attention, thus improving clinical management of flocks. Challenges in ovine medicine include the definition of risk factors on farms, the design control strategies and the enhancement of productivity and livestock welfare. Responding will improve animal health and reduce financial loss.

The high abundance proteome of sheep serum was identified and its usefulness in the biomarker discovery was described, suggesting novel scenarios in investigation of both physiological and pathological conditions.

## Results

### 2-DE sheep serum maps

**Figure 1:**
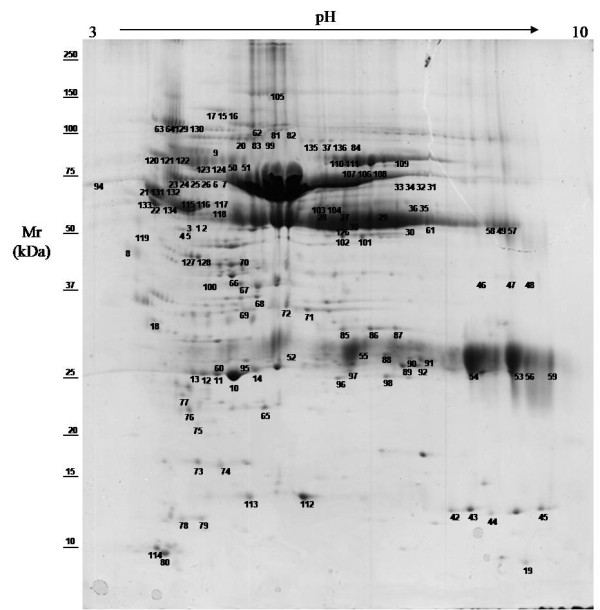
**2-DE reference map of sheep serum pH 3–10.**Representative 2-DE map of sheep serum obtained by performing the first dimension (IEF) on IPG strips pH 3–10 and the second dimension on 9 –16 % gradient SDS-PAGE gels. The protein spots were visualized by Coomassie blue staining. The indicated spots were excised from gel and identified by MALDI-TOF MS and MS/MS. The results of MS identification are reported in Table 1. Mr standard values are indicated on the left.

**Figure 2:**
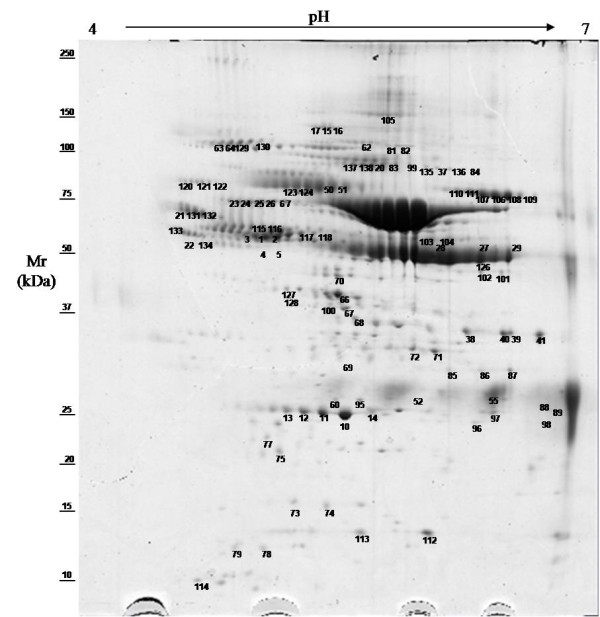
**2-DE reference map of sheep serum pH 4–7.** Representative 2-DE map of sheep serum obtained by performing the first dimension (IEF) on IPG strips pH 4–7 and the second dimension on 9 –16 % gradient SDS-PAGE gels. The protein spots were visualized by Coomassie blue staining. The indicated spots were excised from gel and identified by MALDI-TOF MS and MS/MS. The results of MS identification are reported in Table 1. Mr standard values are indicated on the left.

This study generated the first reference 2-DE gel maps of sheep serum using pH 3–10 (Figure 1) and pH 4–7 (Figure 2) IPG strips, and 9–16 T% SDS-PAGE. The pH 3–10 IPG showed more than 250 spots, most of which were identified as predominant, highly abundant proteins like albumin, and immunoglobulins and their isoforms and fragments. Some had a horizontal train aspect which is typical of glycosylated/phosphorylated proteins. Common serum proteins that have been detected in other species such as humans, horses, chickens, and cattle were observed. Sheep serum protein profiles were improved using pH 4–7 IPG strips. Maps in this pH range had more than 350 spots. Most serum proteins had a pI in the acid range, as can be seen in Figure 2 and most acid proteins showed better resolution in gels covering pH 4–7.

Our preliminary study was designed to detect less abundant serum proteins. We used common strategies for protein depletion in sheep serum like Aurum™Affi-Gel Blue kit (Bio-Rad), the ProteoPrep immunoaffinity albumin and IgG depletion kit (SIGMA), Albumin & IgG Depletion SpinTrap™ (GE Healthcare). Unfortunately, abundant proteins were not significantly depleted, while less abundant proteins were reduced or sometimes eliminated (data not shown) [24]. This was unsuccessful probably because the kits used were designed for human samples. As the methodology was not suitable for our aims in this study it was abandoned and we focussed on non pre-fractionated samples.

MS analysis was performed to identify protein spots from the reference 2-DE gel, and determine protein spots that changed in diseased sheep or during the peripartum period. Protein spots were excised, in-gel digested with trypsin, and peptides were analyzed by MS. Overall, 250 protein spots were analyzed, and 138 were identified, corresponding to 42 proteins. Details of MS protein spot identification are shown in Table 1. In one protein spot (52), two proteins were present. Many proteins were identified in more than one spot. In particular, serum albumin was identified in 40 spots. The diverse protein species were probably due to post-translational modifications, truncations or degradation of mature proteins.

**Table 1:** List of identified sheep serum proteins

**SPOT N° ^(a)^**	**NAME**	**ACCESSION CODE^(b)^**	**Theoretical Mr/pI**	**Experimental Mr/pI ^(e)^**	**# peptides MS**	**# total peptides MS/MS**	**Mascot SCORE^(c)^**	**Orthologue from^(d)^**
1	α 1 Antitripsin	A1AT_SHEEP	45.984/5.83	63.0/4.47	17	2	279 m	
2	α 1 Antitripsin	A1AT_SHEEP	45.984/5.83	62.8/4.55	14	10	311 e	
3	α 1 Antitripsin	A1AT_SHEEP	45.984/5.83	63.7/4.40	16	9	249 e	
4	α 1 Antitripsin	A1AT_SHEEP	45.984/5.83	50.5/4.43	7	2	45 m	
5	α 1 Antitripsin	A1AT_SHEEP	45.984/5.83	50.4/4.52	9	3	99 m	
6	α 1B Glycoprotein	A1BG_BOVIN	53.553/5.30	74.1/4.39	16	5	176 m	*Bovine*
7	α 1B Glycoprotein	A1BG_BOVIN	53.553/5.30	73.3/4.48	9	4	146 m	*Bovine*
8	α 1 acid Glycoprotein	A1AG_BOVIN	23.182/5.62	46.1/3.35	6	2	82 m	*Bovine*
9	Amine Oxidase	AOCX_BOVIN	84.756/5.57	90.1/5.10	19	5	161 m	*Bovine*
10	Apolipoprotein A1	O02762_SHEEP	66.173/4.61	25.2/5.24	18	8	256 m	
11	Apolipoprotein A1	O02762_SHEEP	66.173/4.61	25.7/5.01	11	3	137 m	
12	Apolipoprotein A1	O02762_SHEEP	66.173/4.61	26.0/4.80	31	16	650 m	
13	Apolipoprotein A1	O02762_SHEEP	66.173/4.61	26.2/4.63	13	2	235 m	
14	Apolipoprotein A1	O02762_SHEEP	66.173/4.61	26.0/5.51	15	8	273 e	
15	Ceruloplasmin	CERU_SHEEP	119.126/5.50	137.2/5.06	16	5	267 m	
16	Ceruloplasmin	CERU_SHEEP	119.126/5.50	137.8/5.15	15	7	279 m	
17	Ceruloplasmin	CERU_SHEEP	119.126/5.50	137.1/5.01	14	6	218 m	
18	Protein kinase	KAPCA_SHEEP	40.589/8.71	32.5/3.66	11	3	95 m	
19	Complement component 3	O97941_BOVIN	17.044/5.25	67/9.38	5	2	81 m	*Bovine*
20	Corticotropin releasing factor	CRFR1_SHEEP	47.558/9.63	93.7/5.46	7	2	62 m	
21	Fetuin Precursor	FETUA_SHEEP	38.680/5.17	69.7/4.02	7	4	157 m	
22	Fetuin Precursor	FETUA_SHEEP	38.680/5.17	56.2/4.04	8	3	116 m	
23	α 1B Glycoprotein	A1BG_BOVIN	53.553/5.30	75.6/4.16	14	6	218 m	*Bovine*
24	α 1B Glycoprotein	A1BG_BOVIN	53.553/5.30	74.8/4.23	12	6	239 m	*Bovine*
25	α 1B Glycoprotein	A1BG_BOVIN	53.553/5.30	74.5/4.26	21	13	366 m	*Bovine*
26	α 1B Glycoprotein	A1BG_BOVIN	53.553/5.30	73.9/4.35	11	2	88 m	*Bovine*
27	Fibrinogen α chain	FIBA_BOVIN	67.012/6.73	56.0/6.31	6	2	81 m	*Bovine*
28	Fibrinogen β chain	FIBB_BOVIN	53.339/8.45	56.6/6.00	14	7	175 e	*Bovine*
29	Fibrinogen β chain	FIBB_BOVIN	53.339/8.45	55.0/6.54	16	6	200 m	*Bovine*
30	Fibrinogen β chain	FIBB_BOVIN	53.339/8.45	55.0/6.70	11	4	132 m	*Bovine*
31	Fibrinogen α chain	FIBA_BOVIN	67.012/6.73	74.4/6.96	5	2	90 m	*Bovine*
32	Fibrinogen α chain	FIBA_BOVIN	67.012/6.73	74.7/6.86	4	2	78 m	*Bovine*
33	Fibrinogen α chain	FIBA_BOVIN	67.012/6.73	74.0/6.72	11	3	73 m	*Bovine*
34	Fibrinogen α chain	FIBA_BOVIN	67.012/6.73	74.4/6.78	5	2	61 m	*Bovine*
35	Fibrinogen α chain	FIBA_BOVIN	67.012/6.73	65.3/6.94	9	2	87 m	*Bovine*
36	Fibrinogen β chain	FIBB_BOVIN	53.339/8.45	65.3/6.81	17	4	130 m	*Bovine*
37	Gelsolin	F2YQ13_SHEEP	80.719/5.59	94.4/5.87	13	4	156 m	
38	Haptoglobin	HPT_BOVIN	44.859/7.83	36.7/6.38	9	4	185 m	*Bovine*
39	Haptoglobin	HPT_BOVIN	44.859/7.83	36.6/6.56	18	5	222 m	*Bovine*
40	Haptoglobin	HPT_BOVIN	44.859/7.83	36.6/6.53	17	4	182 m	*Bovine*
41	Haptoglobin	HPT_BOVIN	44.859/7.83	36.6/6.66	7	2	76 m	*Bovine*
42	Hemoglobin β	HBB_SHEEP	16.073/6.75	12.7/7.82	23	8	516 m	
43	Hemoglobin β	HBB_SHEEP	16.073/6.75	12.6/8.24	21	7	428 m	
44	α globin chain	Q28745_SHEEP	15.178/7.94	12.2/8.70	17	11	306 m	
45	α globin chain	Q28745_SHEEP	15.178/7.94	12.2/9.72	18	10	491 m	
46	Ig λ -1 chain	CAA494511	51.564/5.65	41.0/8.69	4	3	141 e	
47	Ig λ -1 chain	CAA494511	51.564/5.65	41.0/8.82	7	5	158 e	
48	Ig λ -1 chain	CAA494511	51.564/5.65	41.0/8.97	7	3	137 e	
49	Ig λ -1 chain	CAA494511	51.564/5.65	51.4/8.65	8	6	150 e	
50	Ig mu chain	AAA513791	53.545/5.10	80.9/5.31	17	12	319 m	
51	Ig mu chain	AAA513791	53.545/5.10	80.9/5.45	16	12	314 m	
52	Apolipoprotein A1	AAI02942	30.276/5.71	26.9/5.68	24	7	399 m	*Bovine*
	IgG α λ-chain	AAU45093	11.328/8.48	26.9/5.68	19	10	337 m	
53	IgG α λ-chain	AAU45093	11.328/8.48	26.6/9.11	14	6	202 m	
54	IgG α λ-chain	AAU45093	11.328/8.48	26.7/8.27	9	3	113 m	
55	IgG α λ-chain	AAU45093	11.328/8.48	27.6/6.39	14	5	286 m	
56	IgG α λ-chain	AAU45093	11.328/8.48	26.4/9.43	11	4	146 m	
57	IgG α λ-chain	AAU45093	11.328/8.48	51.2/8.78	7	2	98 m	
58	Ig λ -1 chain	CAA494511	51.564/5.65	51.1/8.69	5	2	68 m	
59	IgG α λ-chain	AAU45093	11.328/8.48	26.1/9.95	13	2	118 m	
60	IgG α λ-chain	AAU45093	11.328/8.48	27.1/5.13	7	3	115 m	
61	Ig mu chain	AAA513791	53.545/5.10	52.4/6.85	8	2	102 m	
62	α Tripsin Inhibitor Chain H4	ITIH4_BOVIN	101.512/6.22	119.4/5.52	7	2	82 m	*Bovine*
63	Muscle Endopin 1b	SPA33_BOVIN	46.326/6.05	126.3/4.18	6	2	88 m	*Bovine*
64	Muscle Endopin 1b	SPA33_BOVIN	46.326/6.05	126.4/4.28	16	4	142 m	*Bovine*
65	Retynol-Binding protein	RET3_SHEEP	27.992/3.49	22.2/5.59	14	6	287 m	
66	Serum albumin	ALBU_SHEEP	69.188/5.80	44.3/5.22	13	8	236 m	
67	Serum albumin	ALBU_SHEEP	69.188/5.80	42.0/5.23	12	6	193 e	
68	Serum albumin	ALBU_SHEEP	69.188/5.80	39.8/5.53	19	10	359 m	
69	Serum albumin	ALBU_SHEEP	69.188/5.80	34.7/5.52	17	7	279 m	
70	Serum albumin	ALBU_SHEEP	69.188/5.80	46.6/5.23	9	4	138 m	
71	Serum albumin	ALBU_SHEEP	69.188/5.80	34.8/5.95	14	8	258 m	
72	Serum albumin	ALBU_SHEEP	69.188/5.80	35.6/5.77	15	6	212 m	
73	Serum albumin	ALBU_SHEEP	69.188/5.80	16.5/4.68	12	5	167 e	
74	Serum albumin	ALBU_SHEEP	69.188/5.80	16.1/5.05	16	7	222 m	
75	Serum albumin	ALBU_SHEEP	69.188/5.80	21.8/4.53	15	9	326 m	
76	Serum albumin	ALBU_SHEEP	69.188/5.80	22.1/4.47	15	10	332 m	
77	Serum albumin	ALBU_SHEEP	69.188/5.80	23.9/4.41	16	9	335 m	
78	Serum albumin	ALBU_SHEEP	69.188/5.80	11.2/4.36	11	5	176 e	
79	Serum albumin	ALBU_SHEEP	69.188/5.80	11.3/4.06	8	2	93 e	
80	Serum albumin	ALBU_SHEEP	69.188/5.80	9.6/3.93	7	4	119 m	
81	Serum albumin	ALBU_SHEEP	69.188/5.80	116.4/5.69	7	2	71 m	
82	Serum albumin	ALBU_SHEEP	69.188/5.80	116.3/5.74	10	2	115 e	
83	Serum albumin	ALBU_SHEEP	69.188/5.80	93.5/5.53	21	11	353 m	
84	Serum albumin	ALBU_SHEEP	69.188/5.80	92.8/6.08	19	8	275 m	
85	Serum albumin	ALBU_SHEEP	69.188/5.80	31.3/6.29	18	6	248 m	
86	Serum albumin	ALBU_SHEEP	69.188/5.80	31.3/6.55	13	5	173 m	
87	Serum albumin	ALBU_SHEEP	69.188/5.80	30.8/6.83	14	7	273 m	
88	Serum albumin	ALBU_SHEEP	69.188/5.80	27.4/6.70	15	6	237 m	
89	Serum albumin	ALBU_SHEEP	69.188/5.80	25.5/6.94	16	6	254 m	
90	Serum albumin	ALBU_SHEEP	69.188/5.80	26.4/7.03	15	8	270 m	
91	Serum albumin	ALBU_SHEEP	69.188/5.80	26.4/7.25	14	5	195 m	
92	Serum albumin	ALBU_SHEEP	69.188/5.80	25.7/7.16	16	5	219 m	
93	Serum albumin	ALBU_SHEEP	69.188/5.80	36.5/6.26	13	7	265 m	
94	Serum albumin	ALBU_SHEEP	69.188/5.80	74.3/3.05	15	7	261 m	
95	Serum albumin	ALBU_SHEEP	69.188/5.80	27.2/5.40	5	2	75 m	
96	Serum albumin	ALBU_SHEEP	69.188/5.80	24.7/6.24	8	4	161 m	
97	Serum albumin	ALBU_SHEEP	69.188/5.80	25.5/6.38	5	2	88 e	
98	Serum albumin	ALBU_SHEEP	69.188/5.80	24.6/6.69	17	10	369 m	
99	Serum albumin	ALBU_SHEEP	69.188/5.80	93.5/5.68	14	7	222 m	
100	Serum albumin	ALBU_SHEEP	69.188/5.80	43.0/4.76	6	2	87 m	
101	Serum albumin	ALBU_SHEEP	69.188/5.80	49.3/6.46	23	13	456 e	
102	Serum albumin	ALBU_SHEEP	69.188/5.80	49.4/6.30	21	12	401 m	
103	Serum albumin	ALBU_SHEEP	69.188/5.80	63.704	15	8	276 m	
104	Serum albumin	ALBU_SHEEP	69.188/5.80	63.9/6.08	22	13	461 m	
105	Serum albumin	ALBU_SHEEP	69.188/5.80	150.2/5.64	21	10	362 m	
106	Transferrin	O18780_SHEEP	66.454/8.29	77.5/6.44	12	4	125 m	
107	Transferrin	O18780_SHEEP	66.454/8.29	77.3/6.33	13	4	145 m	
108	Transferrin	O18780_SHEEP	66.454/8.29	76.9/6.54	8	3	142 e	
109	Transferrin	O18780_SHEEP	66.454/8.29	82.1/6.66	18	8	279 m	
110	Transferrin	O18780_SHEEP	66.454/8.29	82.8/6.22	6	2	60 m	
111	Transferrin	O18780_SHEEP	66.454/8.29	82.1/6.32	6	3	117 m	
112	Transthyretin	TTHY_SHEEP	15.770/5.59	13.0/5.87	15	9	375 e	
113	Transthyretin	TTHY_SHEEP	15.770/5.59	13.1/5.42	18	14	479 e	
114	Apolipoprotein AII	APOA2_BOVIN	11.201/7.80	10.0/4.03	5	2	77 m	*Bovine*
115	VitD Binding Protein	VTDB_BOVIN	53.342/5.36	64.7/4.48	5	2	104 m	*Bovine*
116	VitD Binding Protein	VTDB_BOVIN	53.342/5.36	64.7/4.56	5	2	46 m	*Bovine*
117	VitD Binding Protein	VTDB_BOVIN	53.342/5.36	63.2/4.86	4	2	40 m	*Bovine*
118	VitD Binding Protein	VTDB_BOVIN	53.342/5.36	62.9/4.92	5	2	59 e	*Bovine*
119	Zn A2 Glycoprotein	ZA2G_BOVIN	33.851/5.11	50.2/3.66	13	7	271 m	*Bovine*
120	Factor XIIa Inhibitor	F12AI_BOVIN	51.723/6.19	88.4/4.06	6	2	82 e	*Bovine*
121	Factor XIIa Inhibitor	F12AI_BOVIN	51.723/6.19	88.1/4.12	6	3	119 m	*Bovine*
122	Factor XIIa Inhibitor	F12AI_BOVIN	51.723/6.19	87.8/4.18	9	3	148 m	*Bovine*
123	Ig mu chain	AAA513791	53.545/5.10	82.8/4.88	13	3	120 m	
124	Ig mu chain	AAA513791	53.545/5.10	81.8/5.01	14	5	152 m	
125	Ig λ -6a light chain	AAB94911	11.718/4.65	24.2/4.87	11	4	135 m	
126	Ig G heavy chain	AAB37381	35.848/6.09	53.8/6.31	7	2	68 m	*Bovine*
127	Paraoxonase 1	Q2KIW1_BOVIN	39.841/5.24	46.8/3.89	9	4	137 m	*Bovine*
128	Paraoxonase 1	Q2KIW1_BOVIN	39.841/5.24	46.6/4.01	8	3	122 e	*Bovine*
129	Muscle Endopin 1b	SPA33_BOVIN	46.326/6.05	126.6/4.38	10	6	192 m	*Bovine*
130	Muscle Endopin 1b	SPA33_BOVIN	46.326/6.05	126.4/4.58	11	7	204 m	*Bovine*
131	Fetuin	FETUA_SHEEP	38.680/5.17	68.1/4.06	8	2	83 m	
132	Fetuin	FETUA_SHEEP	38.680/5.17	67.7/4.12	10	4	172 m	
133	Fetuin	FETUA_SHEEP	38.680/5.17	66.2/4.01	12	7	209 m	
134	Fetuin	FETUA_SHEEP	38.680/5.17	56.2/4.42	11	5	163 m	
135	Gelsolin	F2YQ13_SHEEP	80.719/5.59	94.4/5.87	10	6	214 e	
136	Gelsolin	F2YQ13_SHEEP	80.719/5.59	92.8/6.08	13	8	245 e	
137	Corticotropin	550074A	45.421/6.51	93.6/5.30	17	11	316 m	
138	Corticotropin	550074A	45.421/6.51	93.9/5.38	10	4	141 m	

a) Number refer to protein spots indicated in Figures.

b) Accession number from SWISS-PROT or NCBI entry is given.

c) MOWSE score required for significant homology (P < 0.05); "m" indicates MALDI-TOF/TOF-MS; "e" indicates CE-ESI-MS.

d) Sheep protein or homologue to.

e) Values for Mr and pI experimentally determined for each spot according to marker proteins.

Many of the non-identified spots led to very good MS and MS/MS data, but returned no hits from the databases. This might be due to the incomplete sheep genome sequencing. Some high quality MS data could not be assigned to sheep proteins, but matched bovine proteins; one MS data set matched a horse protein. The identification of proteins in other organisms is because proteins are highly conserved between sheep and cows. Thus, protein spots contain corresponding orthologs in sheep serum.

### Differentially expressed proteins

We tested the usefulness of 2-DE maps of sheep serum, and the efficiency of the proteomic approach in demonstrating variations in serum protein patterns during early stage disease or particular physiological states. As described in the Methods section, we studied serum protein changes in sheep with RTB.

**Figure 3:**
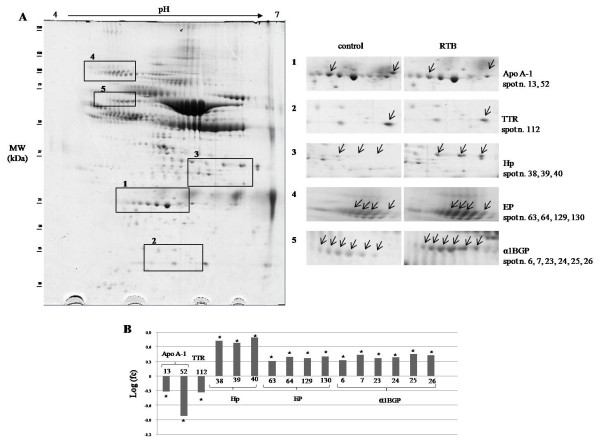
**Differential 2-DE analysis of serum in sheep with RTB. A**) 2-DE serum sheep map and expanded view of the gel portions including the deregulated proteins. Spot proteins differing by > 2 fold (P < 0.05) in sheep with RTB compared to healthy sheep (control) were indicated. Area 1: spots identified as Apo A-1; Area 2: spots identified as TTR; Area 3: spots identified as Hp; Area 4: spots identified as EP; Area 5: spots identified as α1BGP. **B**) Histograms representing log fold change (calculated as a ratio of average density spots in RTB vs average density spots in healthy controls). *: Spots with fold change more than 2 or less than 0.5 with statistically significant P < 0.05.

Serum protein profiles from healthy sheep and sheep affected by RTB are compared in Figure 3A. Overall, 17 protein spots exhibited a fold change (fc) (calculated as a ratio of average density spots in RTB vs average density spots in healthy controls) that was more than 2 or less than 0.5 with statistically significant P < 0.05 (Figure 3B).

In diseased animals we observed a reduction in the two spots corresponding to apolipoprotein A-1 (area 1). A significant decrease was observed for a spot identified as transthyretin (area 2). In contrast, the intensity of three spots identified as haptoglobin was on average five-fold higher (area 3). Significant increases were detected in four spots corresponding to endopin 1b (area 4) and in six spots identified as alpha1B glycoprotein (area 5).

**Figure 4:**
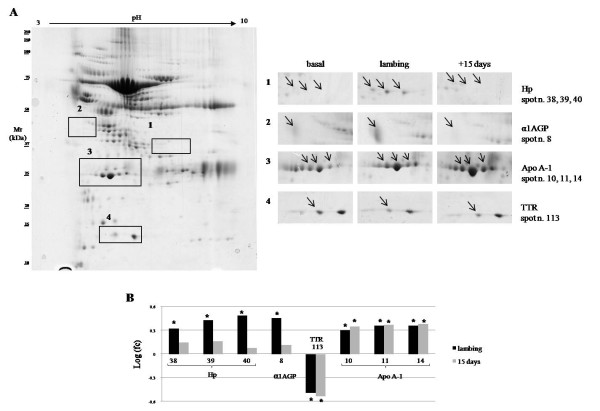
**Differential 2-DE analysis of sheep serum during peripartum.A**) 2-DE serum sheep map and expanded view of the gel portions including the deregulated proteins during peripartum. Spot proteins differing by > 2 fold (P < 0.05) in sheep at lambing (lambing), 15 days after lambing (+15 days) compared to sheep two months before lambing (basal) were indicated. Area 1: spots identified as Hp; Area 2: spots identified as α1AGP; Area 3: spots identified as Apo A-1; Area 4: a spot identified as TTR. **B**) Histograms representing log fold change (calculated as a ratio of average density spots at lambing and +15 days vs basal). *: Spots with fold change more than 2 or less than 0.5 with statistically significant P < 0.05. Black and grey bars correspond to lambing vs basal and +15 vs basal respectively.

We also compared the serum profiles of pregnant healthy sheep two months before the expected delivery date (basal), at lambing, and 15 days after delivery (+ 15 days) (Figure 4A). Fold change was calculated as a ratio of average density spots at lambing and +15 days vs basal (Figure 4B). As above, spots with fc more than 2 or less than 0.5 with statistically significant P < 0.05 were accepted.

Modifications of acute phase proteins were observed. The intensities of three spots identified as haptoglobin were significantly higher at lambing than two months before delivery (area 1). A similar trend was found for alpha-1-acid glycoprotein, which increased at lambing but returned to basal levels within two weeks (area 2). A transthyretin spot intensity before parturition (basal), was higher than at lambing and two weeks after delivery (area 4). Surprisingly, we found that three apolipoprotein A1 spots increased at lambing and after 15 days (area 3).

## Discussion

This paper reports the first proteomic application of 2-DE combined with MS analysis to characterise the total protein profile of sheep serum. Our 2-DE gels can be considered the first reference maps of sheep serum. Most of the serum proteins that are usually used as animal clinical biomarkers were identified. Even though specific new biomarkers are needed for the management of the main diseases that affect sheep, this farm species has been investigated less than others, such as cattle [1-4]. In some cases, therapeutic protocols and diagnostic range values for sheep are borrowed from closely related species such as other ruminants. 2-DE gel reference maps of human, rat, bovine, swine, chicken and horse have been defined [1-7,11,12] highlighting species-specific differences in serum protein profiles.

In the present study, many of the proteins we identified are found in different species, with different pI and molecular weights, and different isoforms and phenotypes [6]. Considering the central role that serum plays in clinical diagnostics, the characterization of 2-DE gel serum sheep protein profiles increases the tools available for ovine medicine, both for identification of new disease markers, and for improved understanding of the physiological and pathological functions of proteins in diseased and healthy sheep.

SELDI-TOF MS has recently been applied to analyse ovine serum [25]. It might be considered complementary to 2-D electrophoresis, because it is able to detect proteins and peptides with a very low molecular mass (< 10–20 kDa). However, many of the proteins with a high molecular weight that could be useful as serum tissue markers remain unrevealed. On the contrary, 2D-electrophoresis cannot detect proteins with a low molecular mass or pI values <4 or >9.

The map presented here is an integral serum 2-DE map which was obtained from whole serum protein content. Serum is rich in proteins such as albumin and immunoglobulin, that can mask less abundant proteins. Nonetheless, we decided to avoid sample pre-treatments, such as dealbumination, that could change the protein composition of our specimens. Our first goal was to provide a map for use in clinical diagnostic and prognostic purposes without pre-fractionating as it might have changed protein quantity or quality. Major serum proteins might be modified in physiological conditions or disease, and consequently, all sample treatments that could mask these changes should be avoided.

Since minor protein components might be involved in metabolism and regulation, one future challenge might be the definition of good depletion strategies or pre-fractioning methods for sheep serum to enhance the identification of low-abundance proteins without altering sample characteristics as has recently been applied to other animal species [26].

The usefulness of the serum reference 2-DE map was confirmed by two different comparative investigations. The first ascertained serum protein pattern differences between healthy sheep and sheep affected by RTB, a common sheep disease. The second one, using a different group of animals, determined the serum protein changes during the peripartum period of four weeks before, and two-three weeks after lambing. The goal was to demonstrate the possible application of this proteomic approach in a mild pathological situation and in the physiological stressful peripartum condition, because in both situations it is important to recognise early signs of sub-clinical conditions.

Similar to previous studies on bovine and swine species [4,6], we observed variations in proteins associated with the acute phase in both cases. Both in RTB affected and in lambing sheep, we saw a decrease in TTR a globular non-glycosylated protein, which is considered to be both a thyroxin- and a holo-retinol-binding protein. TTR is synthesized by different tissues such as the liver, retina, pancreas and placental trophoblast [27]. It performs a multiplicity of biological functions [28]. In particular, it plays a major and critical role in transport of maternal thyroid hormone to the developing fetus [27]. TTR involvement in various central nervous system disorders, diabetes melitus, preclampsia has been described [29-31]. Reduced TTR serum concentrations are often associated with acute phase response, which may be due to inflammation, trauma or other disorders like malnutrition [32,33]. A decrease in TTR during inflammation has been reported as an effect of proinflammatory cytokines such as IL-1 and IL-6 [34]. These findings also explain the decrease in Apo A-1 that we observed in serum from sheep affected by RTB. Apo A-1 is the major protein fraction of high-density lipoproteins, and is considered a negative acute-phase protein [35,36]. During acute inflammation, IL-6, IL-1β, and TNF-α upregulate the expression of the pentraxins (C-reactive protein and serum amyloid-P), which in turn inhibit the synthesis of Apo A-1 [37].

In contrast, we observed a significant increase in Apo A-1 after lambing, which can be explained by the effect of oestrogens. In females, these hormones enhance Apo A-1 levels. In sheep and other ruminants, delivery is induced by an increase in biosynthesis of oestrogens related to progesterone, and an enhancement of the oestrogen/progesterone ratio [38]. A similar situation occurs in humans, but not in other animal species such as horses, and this again confirms the necessity to determine species differences. The above considerations also confirm that some proteins of the acute phase have other functions more important in specific situations. The hormonal changes that occur during lambing and influence lipid transport for lactation are the probable causes of the observed increase in Apo A-1. In sheep affected by RTB, Apo A-1 behaves as a negative acute-phase protein. In these animals, the decrease in negative acute phase proteins TTR and Apo A-1, was related to the increase in Hp [39], a serum protein with several functions. Its main role is to bind free haemoglobin to allow hepatic recycling of haeme iron, and to prevent iron loss and renal damage. As a haemoglobin scavenger, this protein reduces circulating iron and inhibits enzymatic and non-enzymatic reactions catalysed by iron [40]. Hp also acts as an antioxidant, has antibacterial activity, and is involved in modulating aspects of the acute phase response [41]. Its blood level is associated with the prevalence and clinical evolution of many inflammatory diseases including infections, degenerative disorders and autoimmune disorders. Hp is considered a major positive acute-phase protein [42], and its increase was more evident in diseased sheep than in lambing sheep.

The appropriate response to lambing, a critical situation with rapid, intense homeostatic changes, depends on good welfare conditions and an adequate nutritional status. Thus, management of this period is crucial for the health of the ewe, and to prevent disease or infections like mastitis [43,44]. Increased α1AGP after lambing confirmed the stress response during delivery. This acute phase protein has been reported to be involved in similar stressful conditions, inflammation or tissue destruction [45-47].

Although variations in Hp, α1AGP and TTR confirm lambing induces an acute phase reaction, the increase in Apo A-1 indicates a metabolic modification may also be taking place. These proteins may be suggested as putative bio-markers to monitor the adaptive response and metabolic changes in peripartum.

However, in both disease and lambing conditions, no changes were found in other acute phase proteins such as alpha1-antitrypsin. In serum from sheep affected by RTB, we also found an increase in EP. This protein belongs to a large family of serine proteinase (elastase, trypsin, plasmin, chymotrypsin) inhibitors that are essential regulators of a wide variety of biological processes [48]. In particular, EP can strongly inhibit elastase. This protease plays a crucial role in tissue destruction and inflammation in numerous diseases, including chronic obstructive pulmonary disease, cystic fibrosis, adult respiratory distress syndrome, and ischemic-reperfusion injury. EP may play a major role in inflammation and during response to infections in which elastase is particularly involved. In bovine serum, EP content is high, suggesting its involvement in the acute phase response, with other protease inhibitors [49]. The increase in RTB sheep serum of α1BGP, a plasma protein related to the immunoglobulin supergene family, is not clear because its biological role is currently unknown. However, it might be involved in the reaction to infection, and it is considered an acute phase protein. It increases in *Mycobacterium bovis* infected cows [50], but decreases in pigs with viral infections [6]. Even if EP and α1BGP are minor acute phase proteins, their trends suggest further studies are needed to clarify their roles in respiratory/infections disease in sheep and to assess their potential use as putative biomarkers.

## Conclusions

Proteomics has helped discover biomarkers for a large number of human diseases. It has rarely been used in sheep but could serve as a tool to identify protein biomarkers that might be useful for recognising subclinical and pathological conditions, certifying welfare and the quality/safety of ovine products. In the present study a proteomic approach based on 2-DE combined with MS revealed the ovine serum protein pattern and identified 42 medium-high-abundance proteins that could potentially serve as biomarkers in early diagnoses and monitoring of ovine physiology and metabolism. Most were acute phase proteins, even though they act as carry and transport proteins in blood, bind hormones, cytokines and other physiologically important compounds, or are released by dead or damaged cells. Consequently, this study provides the first requisite for proteomic application in ovine medicine and the basis for future investigations that will be designed to specifically assess protein variations in physiological and pathological conditions. Potential applications of our proteomic approach were established by protein variations found in ewes with mild disease and in peripartum period. The road towards a proteomics-based diagnostic test in sheep is still long. This step has to be followed by other phases and markers, sensitivity and specificity need to be tested and established after appropriate validation before efficacy can be assessed using different diagnostic methodologies.

## Methods

### Animals

Two-to-three-year-old nulliparus Comisana ewes were used. All animals were kept at the Didactic and Research estate of the Faculty of Veterinary Medicine, Perugia University, and received the same diet.

Ten healthy sheep were used to generate the reference 2-DE gel maps and as a control group (control).

Seven sheep with signs and symptoms of mild rhino-tracheo-bronchitis were diagnosed by clinical findings and objective examination. They showed increased respiratory rate, coughing, thick nasal discharge, and dyspnoea.

Seven pregnant healthy sheep two months before the expected delivery date (basal), at lambing (lambing), and 15 days (+15 days) after parturition were used to study protein changes during peripartum.

The control and pregnant healthy sheep were selected based on the results of individual physical examination, which included body condition score, clinical evaluation of cardio-pulmonary function, feeding history, a close examination of nose, mouth, eyes, neck and skin, and palpation of the udders to determine if they were healthy and functional [51].

Serum was obtained from blood samples, collected from sheep by veterinary staff, which had already been used to develop routine clinical or health management practices in the flock, following the guidelines of Animal Care and Use Committee of the University of Perugia. Moreover, this study was carried out in accordance with the EU directive 2010/63/EU for animal experiments.

Serum samples, obtained after centrifugation, were mixed with inhibitor protease cocktail (SIGMA) and stored at −80°C.

### 2-D electrophoresis and image analysis

Serum samples containing 800 μg of total proteins (about 10–15 μL) were treated with equal volume of 2.3% DTE, 2% CHAPS at 95°C for 5 min, and diluted in IEF sample buffer (final volume 350 μL) containing 8 M urea, 2 M thiourea, 2% CHAPS, 30 mM Tris, 100 mM DTT, 0.8% ampholytes (Bio-Rad) and bromophenol blue traces. IPG strips (17 cm, pH 3–10 or 4–7) (Bio-Rad) were rehydrated for 12 h at 50 V. Protein concentration was estimated by using the Bradford assay (Bio-Rad) according to the manufacturer 's instructions. Proteins were focused using a Protean IEF Cell (Bio-Rad) at 20°C, with low initial voltage followed by a voltage gradient from 10000 Vh to 95000 Vh, with a limiting current of 50 μA/strip. After focusing, proteins were reduced by incubating the IPG strips with 5 mM tributylphosphine in 10 mL of equilibration buffer (375 mM Tris–HCl pH 8.8, 6 M urea, 20% w/v glycerol, 2% w/v SDS) for 14 min, and alkylated with 2.5% w/v iodoacetamide in 10 mL of equilibration buffer for 14 min. Electrophoresis in the second dimension was carried out in gradient slab polyacrylamide gels (9–16% T) (180 x 240 x 1 mm) in a Protean XL Multi-cells apparatus (Bio-Rad), hosting six gels simultaneously. IPG strips were laid on top of the gels with 0.5% agarose in the cathode buffer (192 mM glycine,15 mM Tris, 0.1% SDS, pH 8.3) containing bromophenol blue. The anode buffer consisted of 375 mM Tris–HCl pH 8.8. Gels were run at 10°C for the first hour at 5 mA/gel and at 10 mA/gel overnight until the dye front reached the bottom of the gel. 2-DE gels were stained with colloidal Coomassie blue G250 (0.12% Blue G-250 (SIGMA), 20% v/v methanol, 10% v/v o-phosphoric acid, 10% w/v ammonium sulphate (SIGMA) and destained in 5% acetic acid [52]. Each sample was analyzed in triplicate.

For image analysis, stained 2-DE gels were immediately scanned using a Bio-Rad GS-800 calibrated densitometer. (Images were analyzed with PDQuest (V7.2) 2-DE gel analysis software (Bio-Rad). Protein spots were detected using the PDQuest software instructions. A reference gel image was created that included all spot information in a matchset, with all the gel images matched using “classic matching” with defined landmarks. All gels compared were matched in one matchset, and the reference gel used to assign spot numbers across all the gels, so that the same spot on each individual gel was assigned to the corresponding spot number for comparison. Isoelectric points and molecular weight were estimated by parallel electrophoresis with a mixture of pI and Mr protein standards (Bio-Rad).

Qualitative and quantitative analysis of protein patterns were conducted by using PDQuest. Master gels were created from four replicated gels from each sheep at each experimental stage. Spots were detected, matched and normalised using the parameter “total of all valid spots”. The threshold for accepting a meaningful variation was a two-fold change, and statistical analysis for significant differences was performed exporting data from PDQuest software to software R by ANOVA. Analysis of variance was followed by the Bonferroni-type multiple comparison to evaluate statistical differences between basal, lambing, and +15 days. (P <0.05 was considered significant).

### MS analysis

Coomassie-stained protein spots were excised and destained with 50% ACN in 50 mM ammonium bicarbonate and dried by addition of ACN and incubation in a speedvac evaporator. Dried gel pieces were rehydrated in 40 mM ammonium bicarbonate and 10% ACN containing 12 ng/μL trypsin (Promega), and incubated for 60 min on ice. The trypsin solution was removed and replaced with 40 mM ammonium bicarbonate and 10 % ACN. After digestion overnight at 37°C, all liquid was collected and the remaining gel pieces extracted twice using 50 μL 5% TFA containing 10% ACN for 30 min with constant shaking. All fluids were combined, dried in a speedvac evaporator and dissolved in 10 μL 10% ACN, 0.2% TFA. About one μL of this peptide solution was applied to a MALDI target (Daltonics) and mixed with two μg CHCA dissolved in 50% ACN, 0.2% TFA as matrix using the dried droplet technique. After crystallization, mass spectra were recorded with an Ultraflex TOF/TOF I mass spectrometer (Bruker Daltonik) operating with FlexControl 2.4, FlexAnalysis 2.4, BioTools 3.0 and WARP-LC 1.1 software. Some protein spots were identified using capillary electrophoresis (CE, P/ACE MDQ-System, Beckman Coulter) coupled to a 3D ion trap mass spectrometer (IT-MS, Esquire3000+, Bruker Daltonik). Briefly, extracted peptides were separated in a 90-cm capillary tube with an inner diameter of 75 μm, containing 0.5 M formic acid and 10% ACN as CE buffer. A stacking method was applied, with 30 nL of 1.5 M NH_3_ loaded into the capillary tube followed by 210 nL of sample, 30 nL 1.0 M formic acid and 20 nL CE buffer. Separation occured in 30 min at 30 kV. The capillary outlet was mounted directly to the electrospray ion source of the IT-MS, and stabilized by addition of 50% isopropanol as sheath liquid (3 μL/min). The ion trap was operated in AutoMSn using a top-2 method and CID mode. Scan range was from 50 to 2200 m/z in MS and MS/MS mode. All obtained spectra were combined and analyzed. To identify proteins, Swissprot and MSDB and NCBI database searches using Mascot 2.0 software were performed considering variable modifications like oxidation of methionine, carbamidomethylation of cysteine, deamidation, and acetylation at the N-terminus. One missed cleavage was allowed and mass tolerance was set to <100 ppm for precursor ions and 0,6 Da for fragment ions. Peptides were considered identified if the peptide ion score was above 25, and proteins were identified by at least two unique peptides. If no sheep protein was identified but an orthologous protein was assigned to the MS and MS/MS spectra this protein was taken as identified and the name and accession No of the orthologous protein was given. Additionally, this protein was checked using a BLAST search.

## Abbreviations

2-DE, Two dimensional electrophoresis; IPG strips, Immobilized pH gradient strips; SDS, Sodium dodecyl sulphate; SDS-PAGE, Sodium dodecyl sulfate polyacrylamide gel electrophoresis; MS, Mass spectrometry; SA, Serum albumin; RTB, Rhino-tracheo-bronchitis; fc, Fold changes; Apo A-1, Apolipoprotein A-1; TTR, Transthyretin; Hp, Haptoglobin; EP, Endopin 1b; α1BGP, Alpha 1B glycoprotein; α1AGP, Alpha-1-acid glycoprotein; TOF, Time of flight; SELDI, Surface-enhanced laser desorption/ionization; IgG, Immunoglobulins; IL, Interleukin; TNF-α, Tumor necrosis factor–α; CHAPS, 3-[(3-Cholamidopropyl)dimethylammonio]-1-propanesulfonate; DTE, 1.4-dithioerythritol; DTT, 1.4-dithiothreitol; IEF, Isoelectrofocusing; pI, Isoelectric point; Mr, Relative molecular mass; ANOVA, Analysis of variance; ACN, Acetonitrile; TFA, Trifluoroacetic acid; MALDI, Matrix-assisted laser desorption/ionization; CHCA, α-cyano-4-hydroxycinnamic acid; 3D, Three dimensional.

## Competing interests

The authors have declared no conflict of interest.

## Authors’ contribution

EC designed and conducted all analyses and wrote the manuscript. LA participated in the design of project, in revising and critical reading of the manuscript. MT participated in the 2D gel analyses. AG participated in critical reading and in revising of the manuscript. IJ participated in critical reading and in revising of the manuscript. FS conducted statistical analysis. ZC participated in the MS analyses. AP designed and conducted MS analyses and participated in writing and revising the manuscript. All authors read, commented on, and approved the final manuscript.
